# Hydrothermal assisted biogenic synthesis of silver nanoparticles: A potential study on virulent candida isolates from COVID-19 patients

**DOI:** 10.1371/journal.pone.0269864

**Published:** 2022-10-06

**Authors:** Fatma O. Khalil, Muhammad B. Taj, Enas M. Ghonaim, Shimaa Abed El-Sattar, Sally W. Elkhadry, Hala El-Refai, Omar M. Ali, Ahmed Salah A. Elgawad, Heba Alshater

**Affiliations:** 1 Clinical and Molecular Microbiology and Immunology Department, National Liver Institute, Menoufia University, Shebin El-Kom, Egypt; 2 Division of Inorganic Chemistry, Institute of Chemistry, The Islamia University Bahawalpur, Bahawalpur, Pakistan; 3 Clinical Biochemistry and Molecular Diagnostics, National Liver Institute, Menoufia University, Shebin El-Kom, Egypt; 4 Epidemiology and Preventive Medicine Department, National Liver Institute, Menoufia University, Shebin El-Kom, Egypt; 5 Department of Chemistry, Turabah University College, Turabah Branch, Taif University, Taif Saudi Arabia; 6 Department of Clinical Pathology, National Liver Institute, Menoufia University Hospital, Menoufia University, Shebin El-Kom, Egypt; 7 Department of Forensic Medicine and Clinical Toxicology, Menoufia University Hospital, Menoufia University, Shebin El-Kom, Egypt; VIT University, INDIA

## Abstract

Till now the exact mechanism and effect of biogenic silver nanoparticles on fungus is an indefinable question. To focus on this issue, the first time we prepared hydrothermal assisted thyme coated silver nanoparticles (T/AgNPs) and their toxic effect on *Candida* isolates were determined. The role of *thyme* (*Thymus Vulgaris*) in the reduction of silver ions and stabilization of T/AgNPs was estimated by Fourier transforms infrared spectroscopy, structure and size of present silver nanoparticles were detected via atomic force microscopy as well as high-resolution transmission electron microscopy. The biological activity of T/AgNPs was observed against *Candida* isolates from COVID-19 Patients. Testing of virulence of *Candida* species using Multiplex PCR. T/AgNPs proved highly effective against *Candida albicans*, *Candida kruzei*, *Candida glabrata* and MIC values ranging from 156.25 to 1,250 μg/mL and MFC values ranging from 312.5 to 5,000 μg/mL. The structural and morphological modifications due to T/AgNPs on *Candida albicans* were detected by TEM. It was highly observed that when *Candida albicans* cells were subjected to 50 and 100 μg/mL T/AgNPs, a remarkable change in the cell wall and cell membrane was observed.

## Introduction

The severe acute respiratory syndrome coronavirus 2 (SARS-CoV-2) is the primary infectious agent of Coronavirus Disease 2019 (COVID-19), a rapidly spreading pneumonia [1]. It is confronting the global public health system. SARS-CoV-2 infects any age with a high impact on the elder people other than SARS-CoV and MERS-CoV [2]. Aside from the pathogenicity of SARS-CoV-2, microbial infections play a crucial role in the occurrence as well as the progression of SARS-CoV-2 infection by making a diagnosis, prognosis, and treatment of COVID-19 more difficult, as well as increasing illness symptoms and mortality [3]. *Candida* strains have been reported in the skin and mucosal dwellers that cause a variety of devastating diseases in immunosuppressed individuals and those who are susceptible [4]. *Candida* species possess numerous virulence factors, including exoenzymes like phospholipase as well as protease, or the capacity to generate the germ tubes and cling to buccal epithelial cells, that aid in the adherence as well as the intrusion of these organisms through the cell membrane, malfunctioning or rupturing [5]. The improper handling of antimicrobial drugs may enhance the prevalence of drug-resistant pathogens [6]. A major cause for the failure of antifungal treatment is low penetration potential to the infection site as well as their side effects [7].

Nanotechnology is one of the promising areas of science that has shown its impact on all scientific fields equally [8, 9]. Nanosized materials, having a size of 20–100 nm, have attracted the attention of researchers because of their propitious characteristics [10]. Engineered silver nanoparticles (AgNPs) have shown their outstanding applications in food-related fields owing to their remarkable antifungal activities [11, 12] and are widely applicable in the biomedical field like cancer photodynamic therapy, biomedical sensing, and molecular imaging as well as drug delivery [13]. Synthesis of nanoparticles via green strategy such as by using plant extract [14], enzymes, phytochemicals etc are preferred mostly as compared to chemically manufactured nanosized material owing to overcome its toxic effect on the environment and living beings [15].

*Thymus vulgaris L*. plant has tremendous medical applications as it was used previously to treat various diseases like stiffening of arteries, urinary tract infections, dyspepsia, pneumonia, respiratory disorders (asthma, bronchitis, or cough), toothache, endocarditis [16–18] and septicaemia due to the presence of biochemical in it and it gains the status of the General Recognized as Safe (GRAS) defined by Food and Drug Administration (FDA) [19]. Thymol in *Thymus vulgaris L*. can destroy the cell membrane of microorganisms [20–22].

In most antifungal treatments, the main biological resistance to drug diffusion is the formation of biofilm that is formed by the aggregation of *Candida albicans* [23, 24]. Drug diffusion decreases due to the formation of biofilm that enhances the cell viscosity [25, 26]. The exact mechanism for antifungal treatment is still unknown for *thyme* coated silver nanoparticles. The present study aims to develop a low cost sustainable hydrothermal method without the addition of any base and with minimum involvement of energy to explore the application and effect of T/AgNPs on membrane damage against *Candida* isolates from Covid-19 patients. Up to our knowledge use of *Thymus valgaris* extract in the hydrothermal approach has not been reported yet.

## Materials and methods

This is a cross-sectional study done using fungal isolates from COVID-19 patients admitted patients of Menoufia Faculty of Medicine, Menoufia University Hospital after patient’s consent (verbal) and approval of Research Ethics Committee of faculty of medicine, Menoufia University (IRB No. 312021/FORE8). Fungal identification Antifungal testing and molecular detection of virulence factors were done in the Microbiological Laboratory, National liver Institute, Menoufia University. All chemicals as well as solvents were of analytical grade and were not purified further.

### Plant material

The *Thymus vulgaris* plant was collected from Egypt. The plant was distinguished at the office of Botany, Faculty of Science, Menoufia University, Shebin El-Kom, Egypt where voucher examples were stored. *Thymus vulgaris* plant was were rinsed with distilled water, weighted, and frozen (−20 °C). After lyophilization (Dura Dry TM μP freeze-drier; −45 °C and 250 mTorr), it was properly stored till used for extraction and synthesis [27].

### Preparation of plant extract

Fresh plant leaves were steeped in 70 percent ethanol/water (100 ml) at room temperature for 7 days after being cleaned with tap water and finally with deionized water. It was then filtered by utilizing Whatman filter paper no. 1 The extract was then warmed to 4 °C and stored for future use [28].

### Synthesis of silver nanoparticles

**Fig 1:**
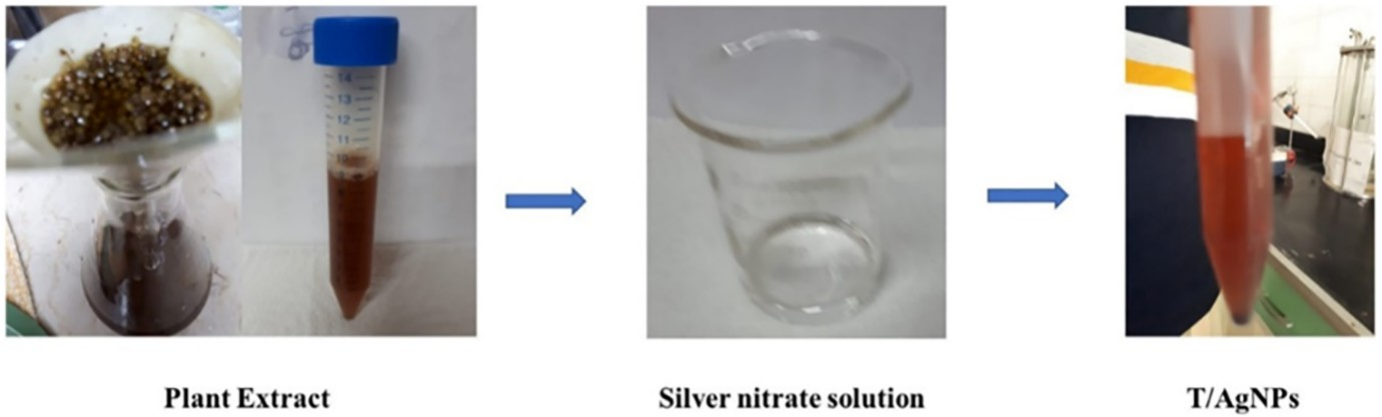
Synthesis of T/AgNPs by plant extract.

Thyme covered silver nanoparticles were synthesized by the modified hydrothermal method [29]. A 25 ml of 0.25 M silver nitrate solution was mixed with 25 ml of plant extract and stirred at 35 °C for 1 h. This solution mixture was transferred into Teflon lined sealed stainless-steel autoclaves and kept in a hydrothermal oven at a temperature of 150 °C for 1.5 h. Then the contents are allowed to cool to room temperature. Pure dark brown colored T/AgNPs were collected by centrifugation at 5000 rpm for 10 minutes. The stable T/AgNPs were dried in an oven at 60 °C for 4 h, ground, and preserved in an airtight bottle (Fig 1).

### Characterization of silver nanoparticles

The analysis was carried out at National Research Centre in Cairo Egypt. A Jasco dual-beam spectrophotometer was used to record the UV–vis spectroscopy estimates (model UV–VIS-NIR 570) operated at a resolution of 2 nm. FTIR measurements were recorded on Perkin Elmer Inc ranging from 4000 nm to 650 nm. Morphological ponders were employed utilizing high-resolution transmission electron microscopy (HRTEM). The HRTEM images were gotten by a JEOL-JEM-2100 version and an atomic force microscope (AFM) was used to determine surface topography and roughness profiles of Nanomaterials using Model 5600LS manufactured by Agilent technology company in the USA. The biological study was implemented in the National Liver Institute, Menoufia University during the period from May 2020 to February 2021 on different samples isolated from COVID-19 patients. The samples were inoculated on Sabrouds dextrose agar (HiMedia, India) and colonies identified by Gram staining, morphology on cornmeal agar, germ tube tests, and chromogenic medium (HiMedia, Mumbai, India), and further identification and antifungal sensitivity were done by the VITEK-2 Yeast identification and AST card (BioMérieux).

### Biological activities

#### DNA extraction

The QIAamp DNA Mini kit (Qiagen, Germany, GmbH) was used to isolate DNA from samples, with certain changes based on the manufacturer’s instructions. A 200 μl sample suspension was incubated at 56 °C for 10 minutes with 10μl proteinase K and 200 μl lysis buffer. Following that, 200 microliters of 100 percent ethanol were poured over the lysate. After that, a centrifuge was used to separate the solution. The nucleic acid was extracted with 100 μl of the kit’s elution buffer.

#### PCR amplification

*Oligonucleotide primer*. The primers used were provided by Metabion (Germany) and are listed in (Table 1). A 25 μl reaction including 12.5 μl of EmeraldAmp Max PCR Master Mix (Takara, Japan), 1 μl of each primer at 20 pmol concentration, 5.5 μl of water, and 5 μl of DNA template was used to test the primers. An Applied biosystems 2720 thermal cycler was used to carry out the reaction.

**Table 1:** Primers sequences, target genes, amplicon sizes and cycling conditions.

Target gene	Primers sequences	Amplified segment (bp)	Primary denaturation	Amplification (35 cycles)			Final extension
				Secondary denaturation	Annealing	Extension	
*ALS3* [30]	CTGGACCACCAGGAAACACT	122	94°C 5 min.	94°C 30 sec.	60°C 30 sec.	72°C 30 sec.	72°C 7 min.
	ACCTGGAGGAGCAGTGAAAG						
*RAS1* [31]	CCCAACTATTGAGGATTCTTATCGTAAA	106	94°C 5 min.	94°C 30 sec.	60°C 30 sec.	72°C 30 sec.	72°C 7 min.
	TCTCATGGCCAGATATTCTTCTTG						
*HYR1* [32]	CGTCAACCTGACTGTTACATC	243	94°C 5 min.	94°C 30 sec.	55°C 30 sec.	72°C 30 sec.	72°C 7 min.
	TCTACGGTGGTATGTGGAAC						
*SAP4* [33]	GCT CTT GCT ATT GCT TTA TTA	394	94°C 5 min.	94°C 30 sec.	49°C 40 sec.	72°C 40 sec.	72°C 10 min.
	TAG GAA CCG TTA TTC TTA CA						

#### Analysis of the PCR products

The PCR products were eluted out using 5V/cm gradients on a 1.5 percent agarose gel (Applichem, Germany, GmbH) in 1x TBE buffer at room temperature. 15 μl of the products were inserted into each gel slot for analysis. The fragment sizes were determined using a general 100 bp ladder (Fermentas, Germany) as well as gelpilot 100 bp ladders. A gel documentation system (Alpha Innotech, Biometra) was used to photograph the gel, and the data was processed using computer software. (Table 3) lists the target genes, primer sequences cycle conditions and amplicon sizes [28].

#### Antifungal susceptibility testing

The antifungal effect of biosynthetic T/AgNPs was evaluated on virulent *Candida albicans* and *non-albicans* isolates using the disc diffusion method using discs; Nystatin 100 U, Clotrimazole 10μg, Fluconazole 10μL, Voriconazole 10μg, Itraconazole10μg, the results obtained were compared for T/AgNPs formed by using the chemical strategy and *Thymus vulgaris*. The disk diffusion method shows the magnitude of the susceptibility of the virulent fungi. The Minimal Inhibitory concentrations (MICs) for fungi were identified as the low quantity at which there is no visible growth can be seen [34].

#### Statistical analysis

The mean and standard error were used to express all the data. To determine the relationship between qualitative variables, the Chi-square test will be performed. When more than 25% of the cells have an anticipated count of less than 5, the Fisher exact test will be applied for 2x2 qualitative variables. When comparing the mean and SD of two sets of quantitative normally distributed data, the Student T-test will be employed, whereas the Mann Whitney test will be used when the data is not normally distributed. When comparing three or more groups with quantitative normally distributed data, the one-way analysis of variance (ANOVA) test will be performed, whereas the Kruskal-Wallis test will be used when the data is not normally distributed. When the P-value is less than 0.05, it is considered statistically significant.

## Results

### UV-vis analysis

**Fig 2:**
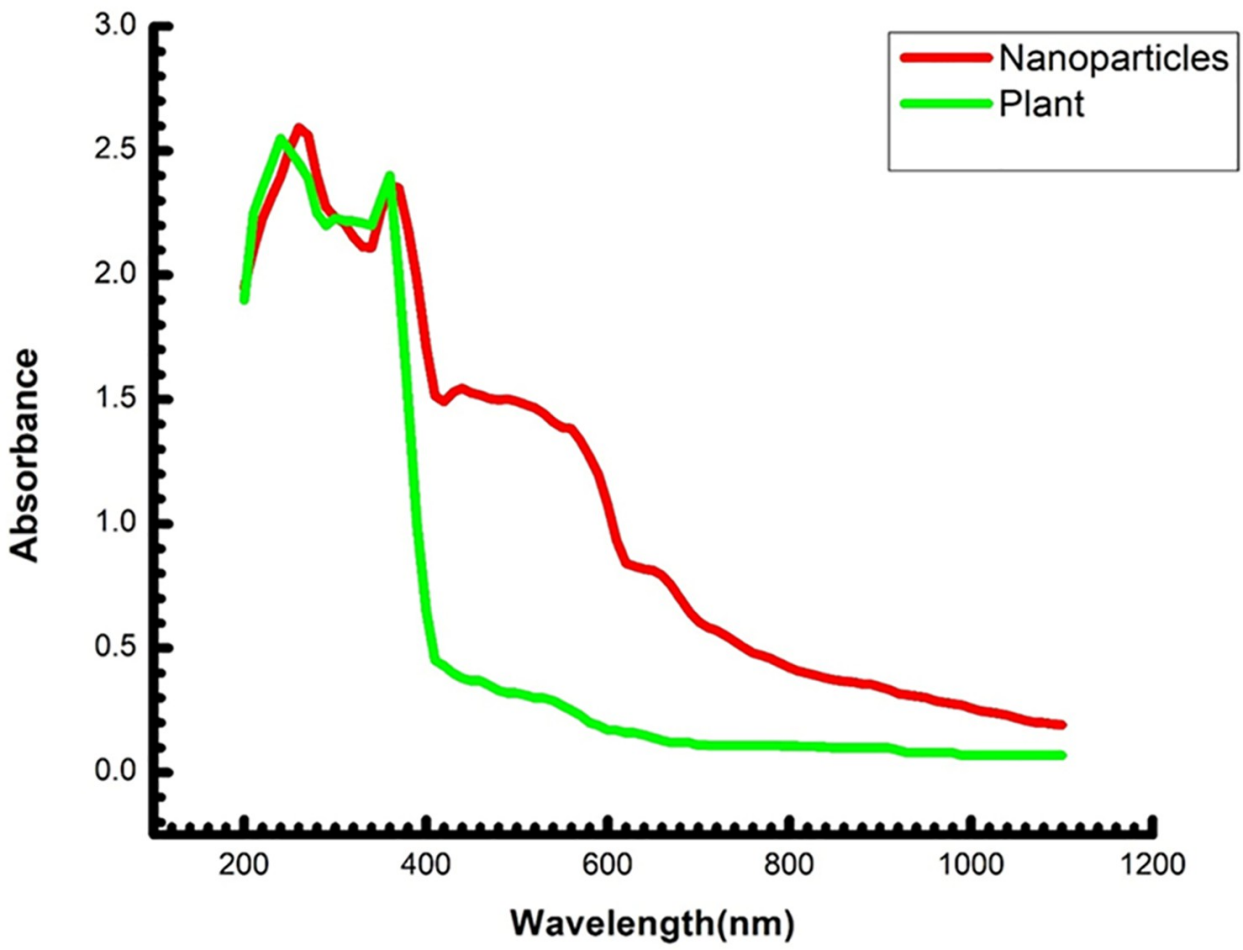
UV-vis spectra of plant extract (green) and silver nanoparticles (red).

The hydrothermal synthesis of T/AgNPs using *Thymus vulgaris* plant extract was detected by visual inspection of the fabricated samples in addition, to monitoring the UV–vis spectra. Alteration of color to yellowish-brown in aqueous solution confirmed the formation of silver nanoparticles [33]. It was observed the color change of colorless solution of silver nitrate to yellowish-brown after 10 min of the addition of extract sample owing to the preparation of silver nanoparticles and this was evaluated by using UV–vis spectrophotometer as depicted in (Fig 2). It was observed that nanoparticles were stable for a long time without being agglomerated. A small band at 438 nm was observed which appeared in the absorption spectra of the fabricated sample (0.1 ml sample). If the concentration of plant extract was increased, the band was positioned from 438 nm to 425 nm.

This band was identified as the absorption by colloidal silver nanoparticles in the visible region (380–450 nm) because of the excitation of surface plasmon vibrations [35]. The intensity of peaks increased and this is an indication of the increase in the concentration of silver nanoparticles [36]. In addition, it was observed that the shifting of the band to a lower wavelength from the maximum absorption wavelength indicated the formation of small-sized Ag nanoparticles with an increased amount of *Thymus vulgaris*. So, it is manifested that there is a successful reduction of silver nanoparticles using *Thymus vulgaris* extract. The stability of silver nanoparticles after various intervals of time was not determined because it is well reported that Thymus Vulgaris mediated synthesis yields stable silver nanoparticles [37–40].

### Fournier infrared analysis (FTIR)

**Fig 3:**
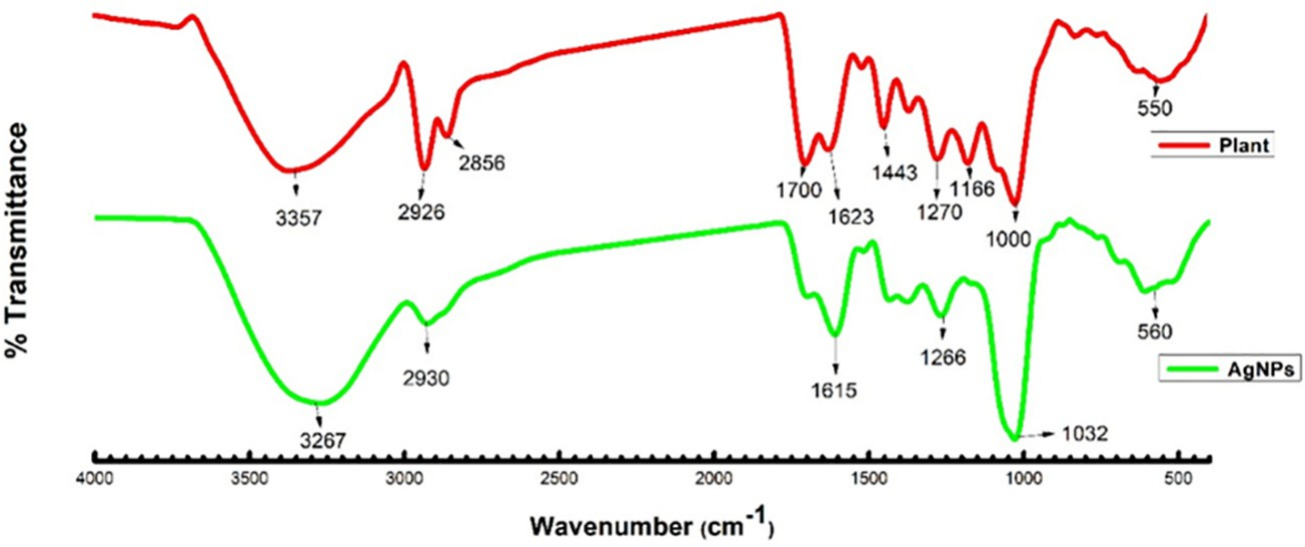
FTIR spectrum of plant extract (red) and silver nanoparticles (green).

The identification of biomolecules that are responsible for the reduction and stabilization of silver nanoparticles was detected by FTIR. *Thymus vulgaris* has a heavy amount of protein and is highly concentrated in amino acids [41]. FTIR measurements for *Thymus vulgaris* extract and reduced silver nanoparticles were depicted in (Fig 3). FTIR transmittance peak at 3357 cm^-1^ was detected for *Thymus vulgaris* extract, which was allotted to OH stretching vibration, which become narrow and shifted to the high region at 3267 cm^-1^. Other bands at 1700 and 1623 cm^-1^ were due to amide I and at 1443 cm^_1^ was assigned to amide II respectively and the peak at 1270cm^-1^ corresponds to amide III [28, 42–44].

The modification in structure is an indication that the reduction, as well as stabilization of silver nanoparticles, advanced through the coordination between the nitrogen atom of the amide group as well as silver ions. It was shown by FTIR measurements that the amide group of protein has a greater capacity to bind metal. The proteins play a significant role as a capping agent as well as preventing the aggregation of nanoparticles.

### High-resolution transmission electron microscopy (HRTEM) analysis

**Fig 4:**
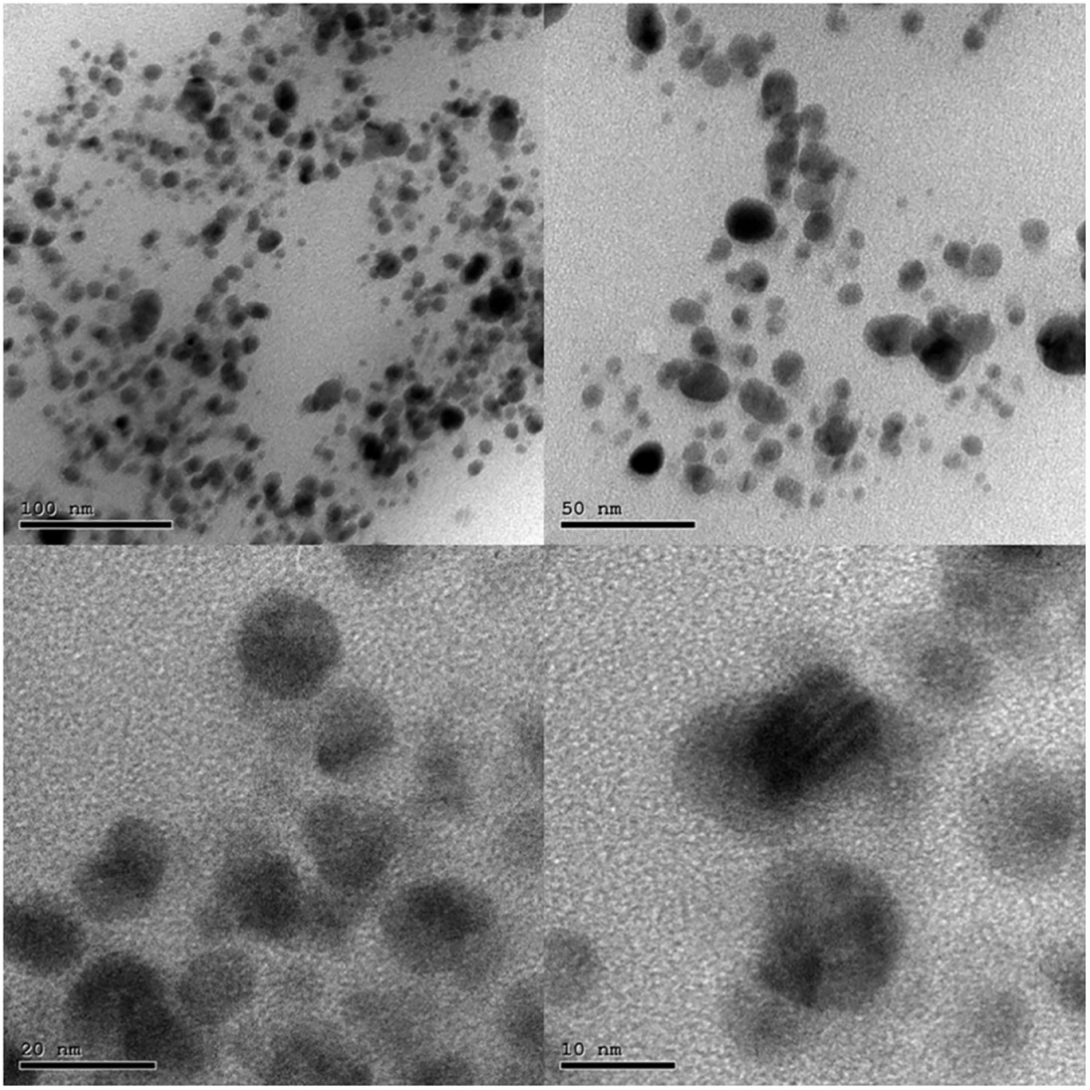
HRTEM image of silver nanoparticles.

TEM measurements are proceeded to evaluate the particle size as well as size distribution for the fabricated nanoparticles (Fig 4). shows HRTEM images registered from the drop coated HRTEM grid of the manufactured Ag nanoparticles. Nanoparticles with agglomeration and spherical shape were depicted in micrographs which are of course due to the aggregation of silver nanoparticles. It is observed that the less dense region around the black spots (nanoparticles) in the HRTEM image could be attributed to the covering of Thymus vulgaris extract.”. At some level of *Thymus vulgaris* extract content, all the nanoparticles are covered, therefore stabilization occurs. So, *Thymus vulgaris* extract is utilized defensive agents to prohibit the aggregation through interaction with initial nanoparticles. and this is in a good agreement explanation of UV–vis spectroscopy results, which is distinguished from well-dispersed silver nanoparticles. The average size of T/AgNPs is determined as 21 nm. The size of each nanoparticle was calculated by using the software in the JEOL-JEM-2100 version and by scale (manually) with reference to the resolution of the image.

### Atomic force microscope (AFM) analysis

**Fig 5:**
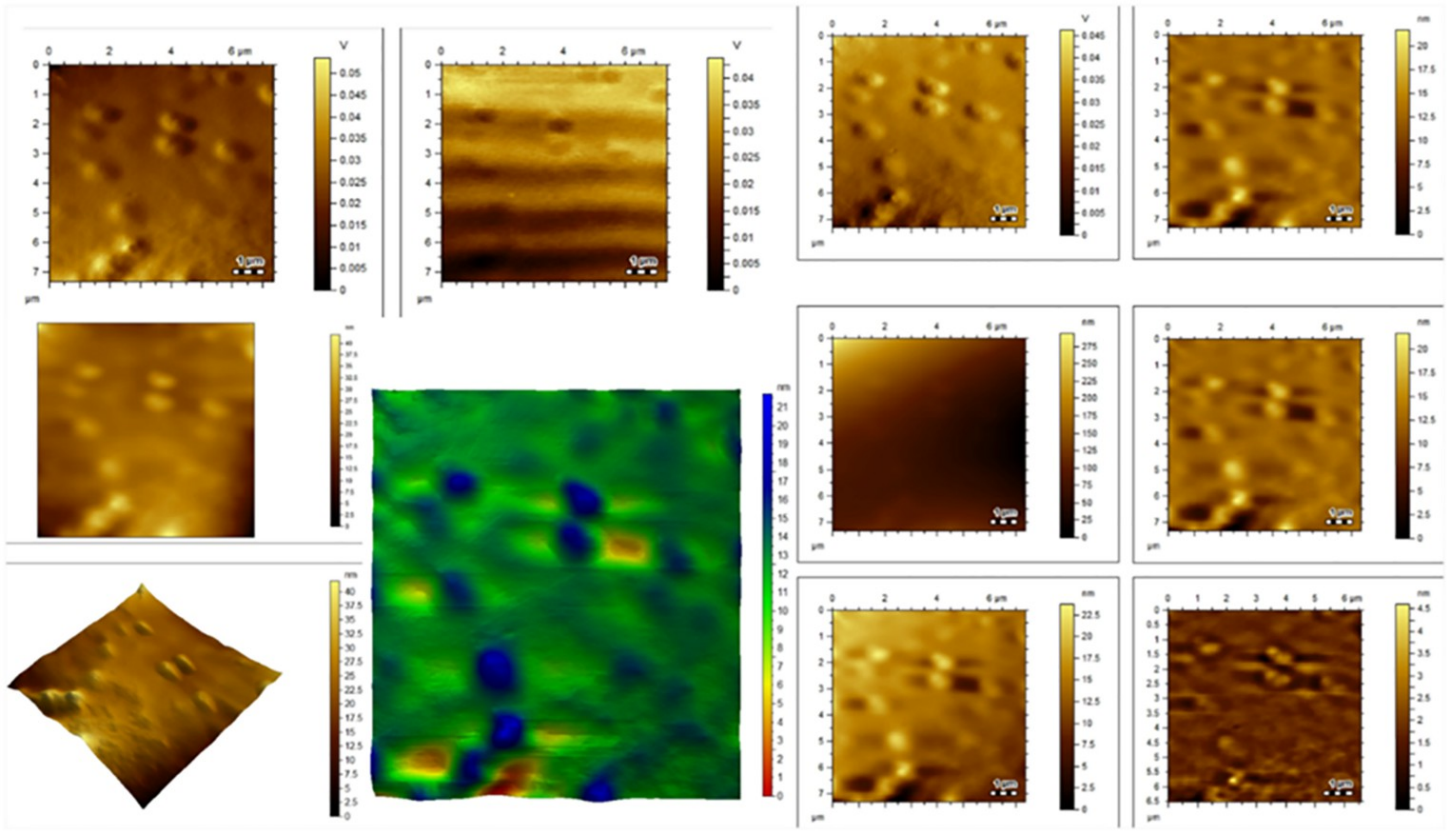
AFM images of *Thyme* based silver nanoparticles (T/AgNPs). “The AFM images display dense aggregates of quasi-spherical AgNPs with an average size of 22 nm. The aggregation shown in AFM images is attributed to the sampling process for AFM leading to dropping a few drops of NPs solution on a silica glass plate and allowing drying to get a thin film of NPs. This will allow the NPs to be clustered together. The AFM images confirm the narrow particle size distribution obtained from TEM images”.

The atomic force of microscopy was utilized for the determination of morphology and agglomeration of silver nanoparticles using *Thymus vulgaris* extract as shown in (Fig 5). Individual particles, as well as clusters of particles, can also be identified with the AFM. Microscope pictures are crucial in research and development initiatives, as well as when fixing quality control problems. The AFM allows for three-dimensional viewing. The vertical, or Z, axis resolution is restricted by the instrument’s vibration environment, whereas the horizontal, or X-Y, axis resolution is limited by the diameter of the scanning tip. Agglomeration of silver nanoparticles biosynthesized on the surface.

### Antimicrobial activity

**Fig 6:**
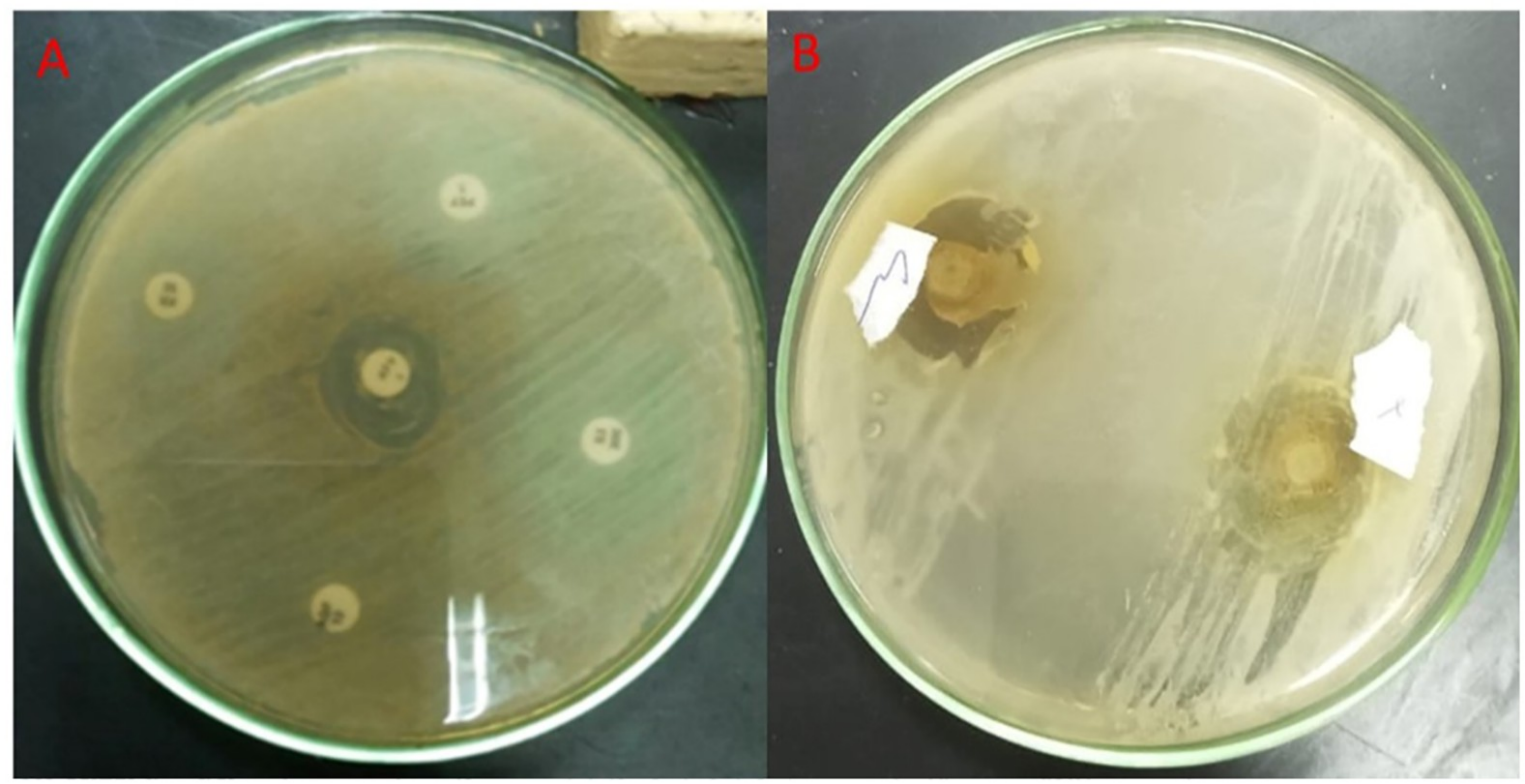
Selected disks show the disk diffusion (antibacterial) activity of silver nanoparticles. (A) Inhibition zone present around the disc dipped in AgNPs and (B) inhibition zone absent around the disc (control).

The antifungal treatment was performed by determining the clear inhibition zone supplied with 0.031–0.5mg/mL of silver nanoparticles. Within this range of concentration, the change and visibility of results were prominent. The development of the *Candida* population was observed to decrease by supplying 0.5 mg/mL of T/AgNPs (Fig 6). The greater inhibition zone (22 mm) was monitored for *Candida albicans*, The MICs value observed for T/AgNPs from 0.125–0.250 mg/mL against *Candida* and bacteria species while MBCs and MFCs value obtained was 0.250 and 0.5000 mg/mL respectively. In the present research T/AgNPs with MIC values ranging from 156.25 to 1,250 μg/mL and MFC values ranging from 312.5 to 5,000 μg/mL against *Candida kruzei*, *Candida glabrata* and *Candida albicans*. It was indicated from MIC and MFC values that T/AgNPs had a greater potential for anticandidal activity. Moreover, Different amount of T/AgNPs ranging from 62.5 to 1000 μg/mL was also applied to check the effect on the development of *Candida tropicalis*, *Candida famata* and *Candida albicans* species, followed by decreased growth of *Candida* species at all concentration of the sample. The *Candida* species growth increases rapidly in the dearth of T/AgNPs. However, when the high quantity of T/AgNPs from 500 to 1000 μg/mL was applied, the *Candida* cells growth was ceased. It has been shown (Fig 6) that the growth inhibition of *Candida* cells was increased with an increased amount of T/AgNPs. The Minimal Inhibitory concentrations (MICs) for fungi were identified as the lowest quantity at which no visual growth was observed. The Minimum fungicidal concentration (MFC) [45, 46] was defined as the lowest drug concentration at which subcultures yielded negative findings or less than three colonies, indicating that >99% of the original inoculum had died.

### Morphological and ultrastructural alteration caused by T/AgNPs

**Fig 7:**
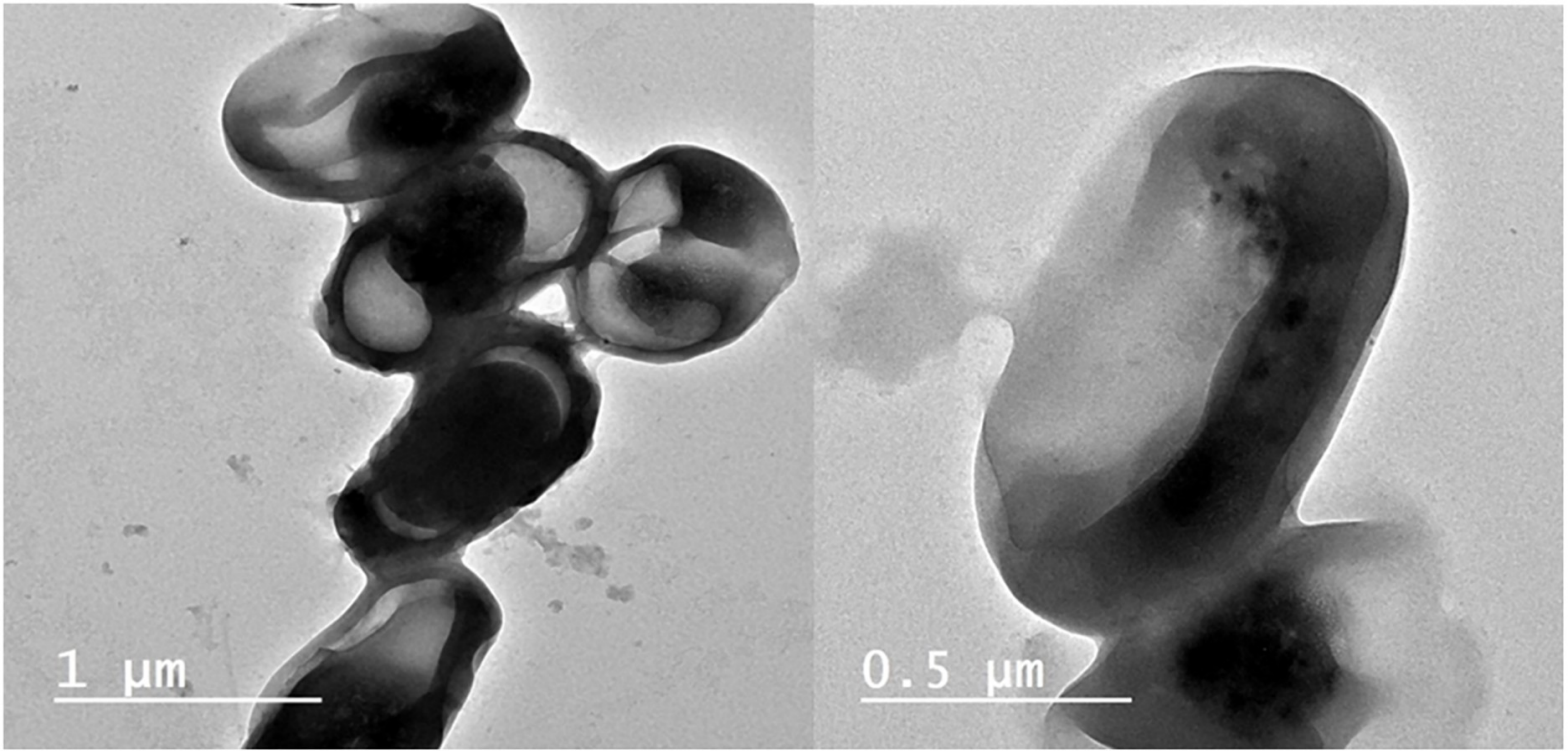
TEM images of the effect of *Thymus vulgaris* silver nanoparticles on *Candida*; destruction of the fungal cell wall with disruption of membrane.

The structural and morphological modifications due to T/AgNPs on *Candida albicans* were detected by TEM. It was detected that when *Candida albicans* cells were subjected to 50 and 100 μg/mL T/AgNPs, a remarkable change in the cell wall and cell membrane was observed (Fig 6). It was found from TEM analysis (Fig 7) that T/AgNPs were adhered not only on the surface of the cell wall and membrane but also penetrate the cell and stored in the cytoplasm followed by the rupturing of the cell wall and destruction of the cytoplasmic membrane (Fig 6). The recent TEM analysis is in good agreement with the previous work on morphological analysis of the effects of T/AgNPs on *Candida* albican*s* [47]. The exact Anticandidal mechanism of nanostructured material is not known. Kim et al studied that the cell membrane and cell wall of the *Candida albicans* species were destroyed by T/AgNPs due to the formation of “pits and holes” on the surface which prevents the process of budding and results in cell death [48]. It is also reported that the antifungal activity of AgNPs is may be due to inhibition of β-glucan synthase and structural modifications of cell walls which lose their proper functioning and are followed by cell death [49]. Furthermore, in other literature, it was reported that T/AgNPs enhance apoptosis in mitochondria, DNA, and nuclear fragmentation, phosphatidylserine externalization as well as the activation of metacaspases in *Candida albicans* and leads to cell destruction due to the build-up of intercellular ROS. Radhakrishnan et al studied that *Candida albicans* cell destruction is not only due to the accumulation of ROS but also the T/AgNPs affect the cellular microenvironment membrane fluidity, structure, and ultrastructure, cellular ergosterol levels, as well as fatty acid composition, particularly oleic acid, which is essential for hyphal morphogenesis [50].

*Candida albicans* isolate is resistant to antifungal agents; Nystatin 100μg, miconazole (10 μg), Clotrimazole (50μg), Flucytosine (25μg) and sensitive to Fluconazole(25μg). taking subcultures on the middle plate showing that the *Candida* isolates are resistant to *Thymus vulgaris* alone (lower part) and sensitive to silver nitrate alone; the zone of inhibition increases on the addition of biologically active silver nitrate solution in the sabrouds agar plate on the right.

### High-resolution Transmission Electron Microscopy (HRTEM)

**Fig 8:**
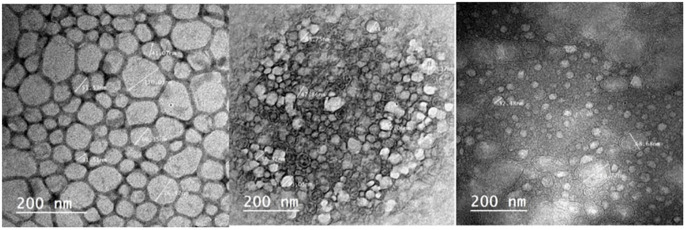
TEM images of SIL (Solid Immersion Lens) showing shrinkage of *Candida albicans* isolates after addition of biologically active silver nitrate particles.

**Fig 9:**
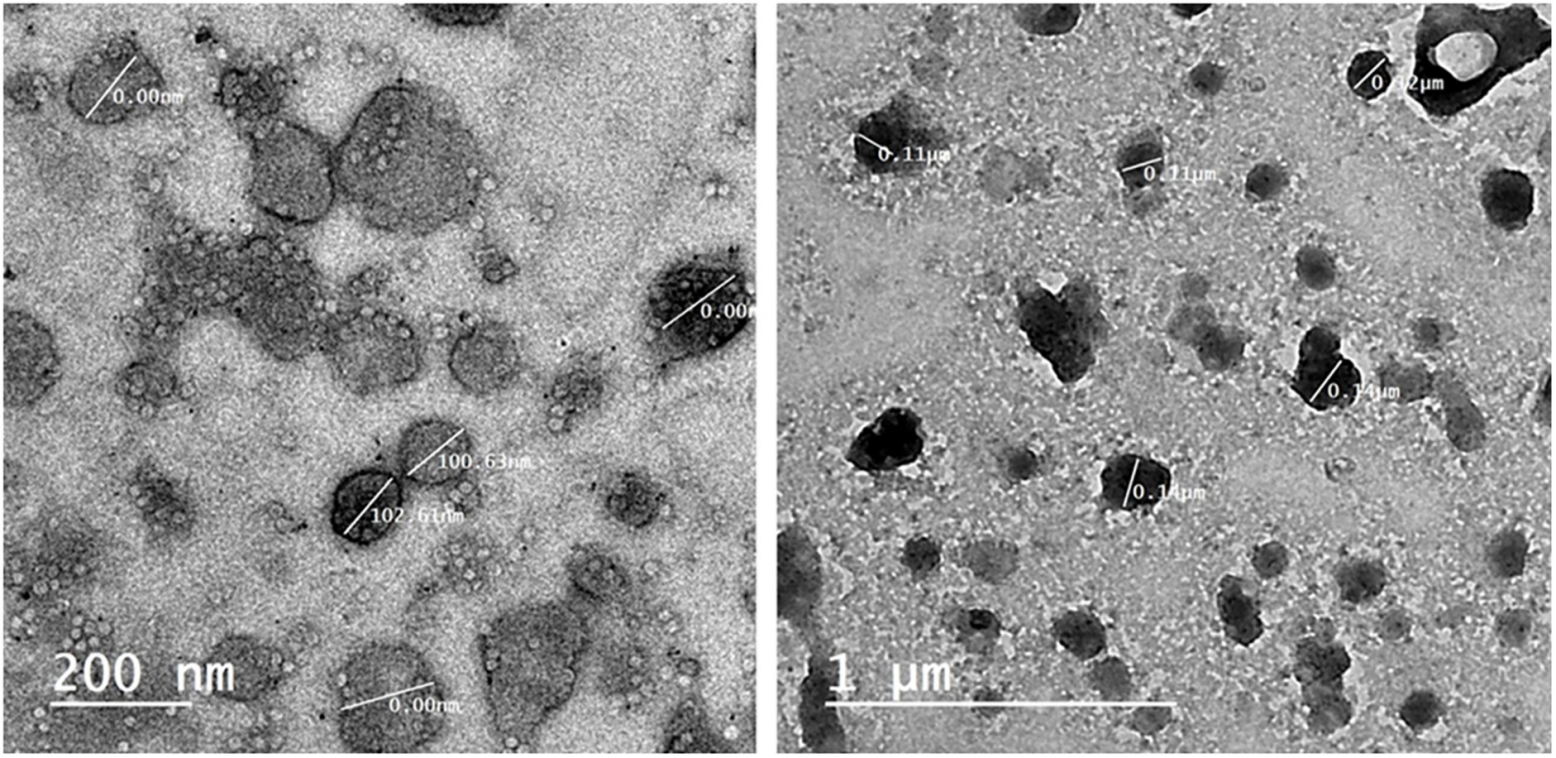
TEM images showing intracellular biologically active T/AgNPs and cellular disruption.

**Fig 10:**
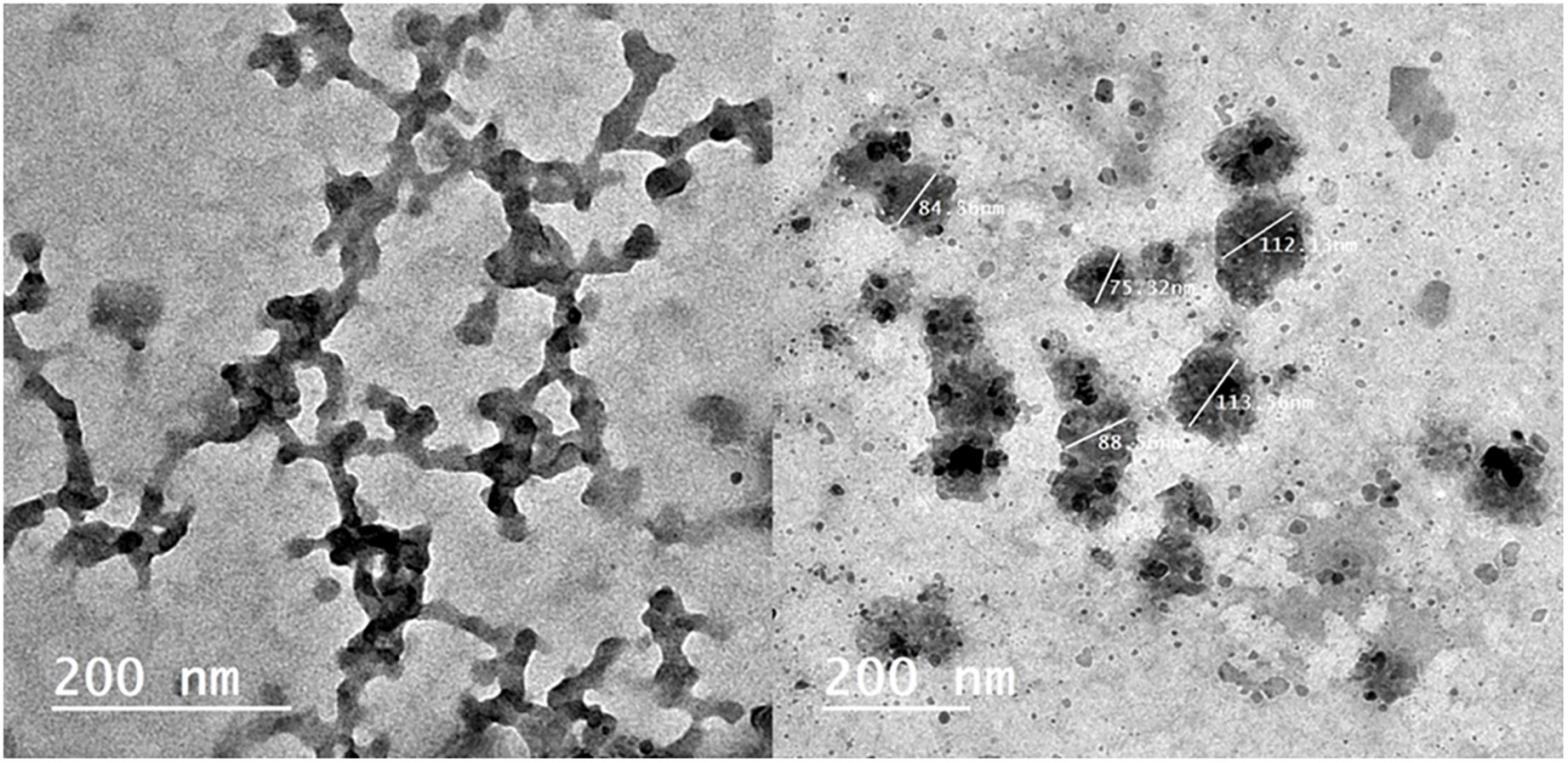
TEM images of the effect of biologically active silver nitrate on *Candida albicans* isolates (image contains disrupted cells).

**Fig 11:**
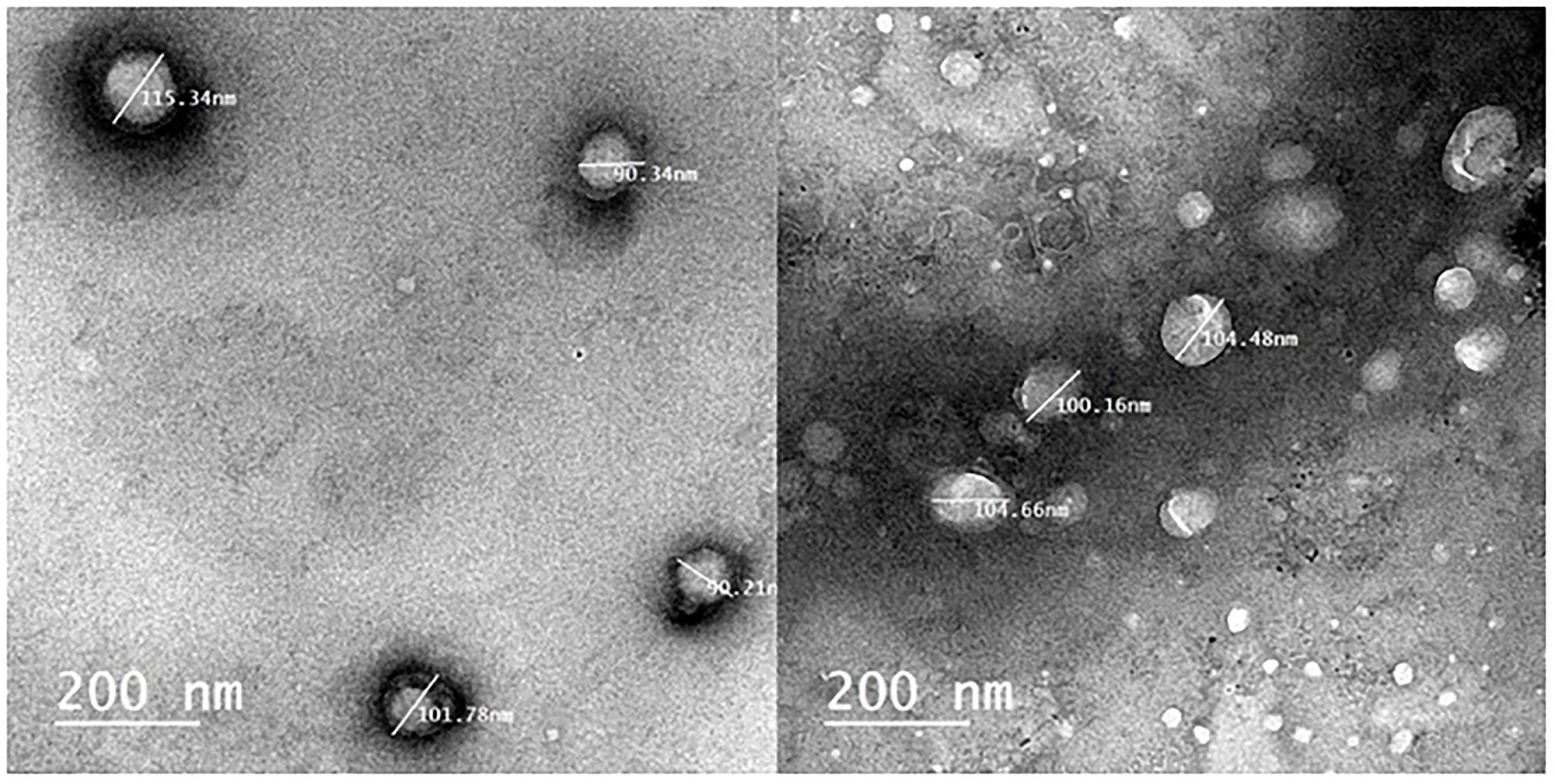
TEM images of intracellular and extracellular distribution of biologically active silver nitrate (measured cells are disrupted).

To visualize the action of biologically active silver nitrate on *Candida albicans* isolates (Figs 8–11) After growth, the fungal cells were centrifuged as well as fixed with 2.5% glutaraldehyde as well as 4% paraformaldehyde (Sigma-Aldrich) and finally contrasted in the solution of 5% uranyl acetate. Dehydration was performed using acetone (Sigma-Aldrich). The samples were analyzed under a transmission electron microscope (Thermo Fisher Scientific, USA).

**Fig 12:**
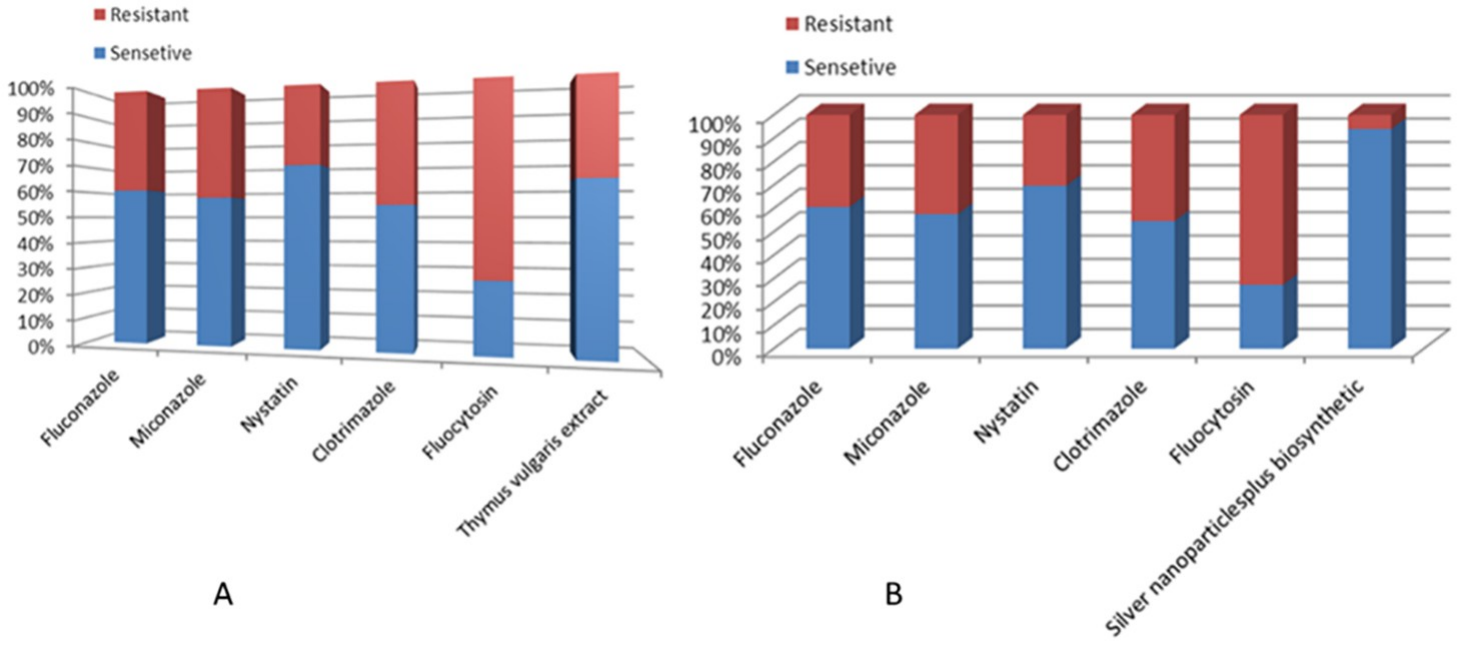
(A) The percentage of resistant isolates to chemical antifungal agents and T/AgNPs. (B) Anti-fungal sensitivity testing of commercial antifungal versus biogenic silver nitrate.

**Fig 13:**
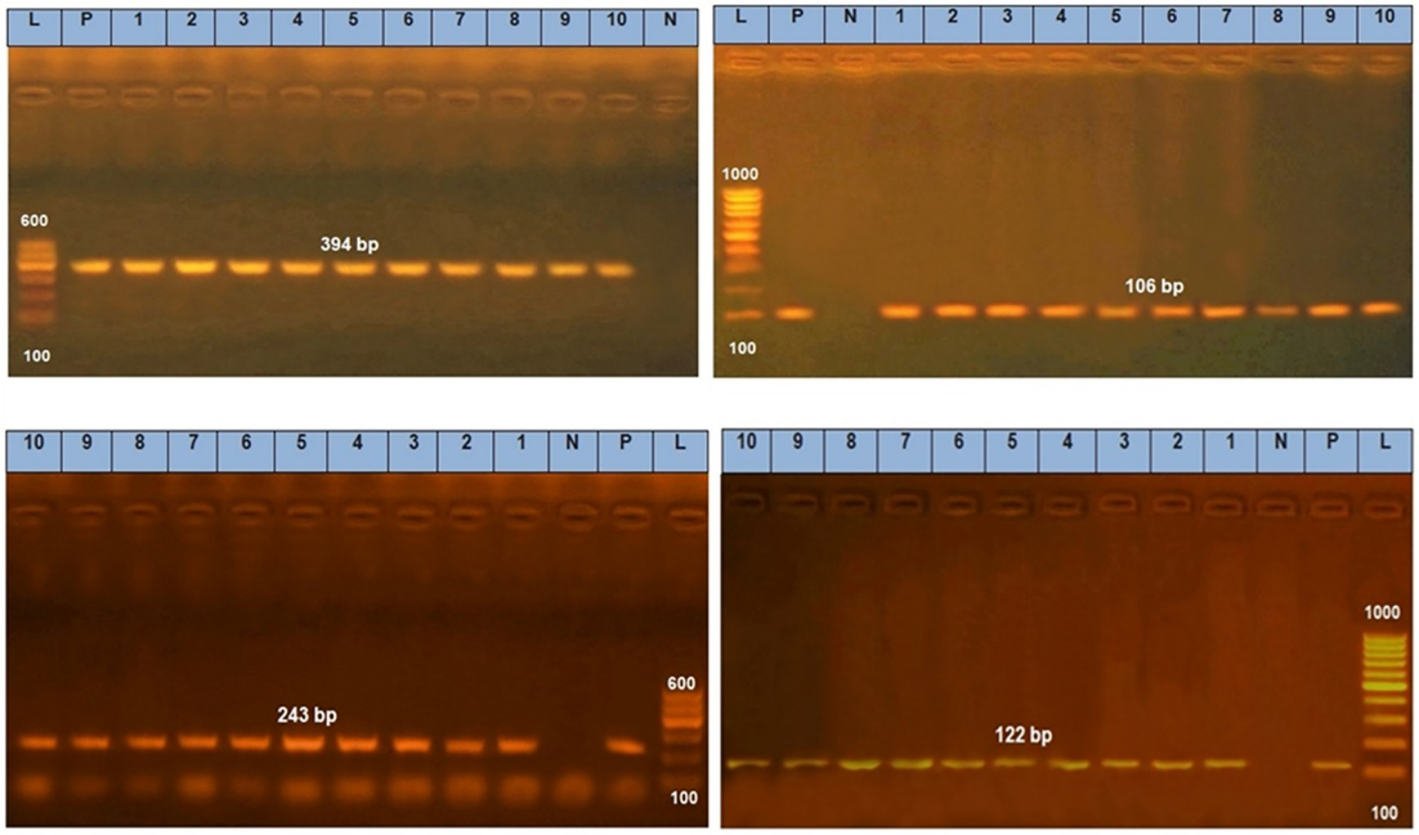
Gel electrophoresis of *Candida* virulence genes, Secreted Aspartic Protease gene (SAP4) was detected at 394 bp, RAS1 was detected at 106 bp, Hyphal-Associated Adhesin (HYR1) gene was detected at 243 bp and agglutinin-like sequence gene (ALS3) was detected at 122 bp.

The sociodemographic features, clinical and laboratory data and prognosis of the studied patients with and without fungal growth are mentioned in (Table 2). *Candida albicans* isolates features as mentioned in (Table 3) regarding antifungal sensitivity and virulence factors. Secreted Aspartic Protease gene (SAP4) was detected at 394 bp, RAS1 was detected at 106 bp, Hyphal-Associated Adhesin (HYR1) gene was detected at 243 bp and agglutinin-like sequence gene (ALS3) was detected at 122 bp (Fig 12). Fig 13 show the effect of *Thymus vulgaris* alone and after union with silver nitrate particles on *Candida albicans* isolates in comparison to other antifungal agents.

**Table 2:** Demographic, clinical-laboratory data, and prognosis of patient groups.

Category		Result		Total	Test	p-value
		Patients without fungal growth N = 14	Patients with fungal growth N = 46			
Sex	Male Female	9 64.3% 5 35.7%	32 69.6% 14 30.4%	41 68.3% 19 31.7%	X^2^ = 0.138	0.749
Age	Mean ± Std Deviation N Median Minimum-Maximum	50.93±6.866 14 52.00 33–60	51.96±7.489 46 52.00 24–66	51.72±7.305 60 52.00 24–66	U = 0.315	0.753
Sample	Ascitic bedsore Blood cannula site sputum Urine	0(0.0%) 1(7.1%) 7(50.0%) 0(0.0%) 5(35.7%) 1(7.1%)	1(2.2%) 2(4.3%) 16(34.8%) 1(2.2%) 15(32.6%) 11(23.9%)	1(1.7%) 3(5.0%) 23(38.3%) 1(1.7%) 20(33.3%) 12(20.0%)	LR = 3.7	0.589
Nasal SARSCOV2PCR	Negative Positive	0 (0.0%) 14(100.0%)	1 2.2% 45 97.8%	1 1.7% 59 98.3%	Fisher = 0.31	0.767
RBS	Mean± SD N Median (Min- Max)	107.29 12.755 14 102.00 98 147	119.09 59.450 46 101.00 82 400	116.33 52.506 60 102.00 82 400	U = 0.861	0.39
CRP	Mean± SD N Median (Min- Max)	50.57 36.091 14 46.00 12 140	53.67 41.095 43 48.00 8 225	52.91 39.633 57 47.00 8 225	U = 0.195	0.845
CBC(WBCS)	Mean± SD N Median (Min- Max)	7.014 5.7567 14 4.150 3.1 19.8	7.941 4.5884 46 6.450 3.1 19.8	7.725 4.8493 60 6.250 3.1 19.8	U = 2.258	0.024*
Lymphocytes	Mean±SD N Median (Min-Max)	68.186±160.9142 14 25.550 6.0–625.0	21.843±12.0892 46 16.800 6.5–54.3	32.657±78.7876 60 18.250 6.0–625.0	U = 1.19	0.234
ESR	Mean±SD N Median (Min-Max)	47.71±30.139 14 51.00 8–85	45.04±30.663 46 40.00 5–110	45.67±30.308 60 40.00 5–110	U = 0.307	0.759
serum ferritin	Mean±SD N Median (Min-Max)	542.93±231.288 14 506.00 320–1150	553.07±291.897 46 519.00 25–1150	550.70±277.113 60 506.00 25–1150	U = 0.105	0.916
D-Dimer	Mean±SD N Median (Min-Max)	23.2857±80.22222 14 2.0000 1.00–302.00	8.6972±44.2214 46 2.0000 .00–302.00	12.1012±54.2977 60 2.0000 .00–302.00	U = 0.74	0.459
LDH	Mean±SD N Median (Min-Max)	543.64±186.578 14 542.50 204–876	640.04±262.926 46 654.00 204–1234	617.55±249.173 60 555.00 204–1234	U = 1.006	0.315
Prognosis	Death Recovery	0 0.0% 14 100.0%	4 8.7% 42 91.3%	4 6.7% 56 93.3%	Fisher = 1.3	0.564

**Table 3:** The sociodemographic, laboratory, antifungal sensitivity, virulence factors in *Candida albicans* versus *Candida* non-albicans isolates.

Category		*Candida*		Total	Test	p-value
		*Candida albican* N = 33	*Candida non albican* N = 10			
Sex	Male Female	24 72.7% 9 27.3%	5 50.0% 5 50.0%	29 67.4% 14 32.6%	X^2^ = 1.8	0.179
Age	Mean Std. Dev. N Median Minimum Maximum	52.12 5.830 33 51.00 43 66	50.00 11.776 10 53.50 24 63	51.63 7.512 43 52.00 24 66	U = 0.403	0.702
Sample	ascitic bedsore Blood cannula site sputum Urine	1 3.0% 2 6.1% 10 30.3% 1 3.0% 10 30.3% 9 27.3%	0 0.0% 0 0.0% 4 40.0% 0 0.0% 4 40.0% 2 20.0%	1 2.3% 2 4.7% 14 32.6% 1 2.3% 14 32.6% 11 25.6%	LH = 2.7	0.745
CORAD	4 5	31 93.9% 2 6.1%	9 90.0% 1 10.0%	40 93.0% 3 7.0%	Fisher = 0.184	0.558
Nasal SARSCOV2PCR	Negative Positive	1 3.0% 32 97.0%	0 0.0% 10 100.0%	1 2.3% 42 97.7%	Fisher = 0.31	1.00
RBS	Mean± SD N Median (Min- Max)	118.00 62.068 33 100.00 87 400	113.60 40.716 10 106.00 82 225	116.98 57.393 43 102.00 82 400	U = 0.202	0.840
CRP	Mean± SD N Median (Min- Max)	55.69 44.397 32 44.00 8 225	52.67 31.149 9 50.00 12 125	55.02 41.512 41 48.00 8 225	U = 0.315	0.752
CBC(WBCS)	Mean± SD N Median (Min- Max)	7.679 4.6798 33 6.400 3.1 19.8	9.440 4.7984 10 6.850 5.4 18.0	8.088 4.7103 43 6.500 3.1 19.8	U = 1.497	0.134
Lymphocytes	Mean± SD N Median (Min- Max)	22.339 12.8928 33 17.200 6.5 54.3	16.700 6.6663 10 15.000 7.0 31.0	21.028 11.9155 43 16.400 6.5 54.3	U = 0.792	0.428
ESR	Mean± SD N Median (Min- Max)	46.67 31.075 33 40.00 5 110	44.60 29.710 10 35.00 6 100	46.19 30.425 43 40.00 5 110	U = 0.217	0.829
serum ferritin	Mean± SD N Median (Min- Max)	569.61 286.640 33 540.00 98 1150	514.80 285.329 10 519.00 25 1150	556.86 283.891 43 540.00 25 1150	U = 0.374	0.708
D-Dimer	Mean± SD N Median (Min- Max)	11.3052 52.19852 33 2.0000 .00 302.00	2.2000 1.03280 10 2.0000 .00 4.00	9.1877 45.73104 43 2.0000 .00 302.00	U = 0.207	0.836
LDH	Mean± SD N Median (Min-Max)	645.88 252.923 33 663.00 204 1234	703.10 292.207 10 732.50 220 1038	659.19 260.066 43 663.00 204 1234	U = 0.504	0.615
Fluconazole	S R	20 60.6% 13 39.4%	3 30.0% 7 70.0%	23 53.5% 20 46.5%	X2 = 2.89	0.089
Miconazole	S R	19 57.6% 14 42.4%	2 20.0% 8 80.0%	21 48.8% 22 51.2%	X2 = 4.337	0.037*
Nystatin	S R	23 69.7% 10 30.3%	4 40.0% 6 60.0%	27 62.8% 16 37.2%	X2 = 2.897	0.089
Clotrimazole	S R	18 54.5% 15 45.5%	3 30.0% 7 70.0%	21 48.8% 22 51.2%	X2 = 1.85	0.174
Fluocytosin	S R	9 27.3% 24 72.7%	1 10.0% 9 90.0%	10 23.3% 33 76.7%	X2 = 1.28	0.257
RAS1	Negative Positive	0 0.0% 33 100.0%	5 50.0% 5 50.0%	5 11.6% 38 88.4%	Fisher = 18.6	0.0001**
ALS3	Negative Positive	0 0.0% 33 100.0%	4 40.0% 6 60.0%	4 9.3% 39 90.7%	Fisher = 14.55	0.002**
Hyr1	Negative Positive	3 9.1% 30 90.9%	7 70.0% 3 30.0%	10 23.3% 33 76.7%	X2 = 15.9	0.0001**
SAP4	Negative Positive	1 3.0% 32 97.0%	4 40.0% 6 60.0%	5 11.6% 38 88.4%	Fisher = 10.2	0.007**
biosynthetic anti-fungal	S R	21 63.6% 12 36.4%	5 50.0% 5 50.0%	26 60.5% 17 39.5%	X2 = 0.597	0.44
Silver nanoparticles plus biosynthetic	S R	31 93.9% 2 6.1%	9 100.0% 0 0.0%	40 95.2% 2 4.8%	X2 = 0.573	1.00
Prognosis	death recovery	0 0.0% 33 100.0%	4 40.0% 6 60.0%	4 9.3% 39 90.7%	Fisher = 14.55	0.002**

*Candida* species, *Aspergillus*, and *P*. *jirovecii* infections were found in critically unwell COVID-19 patients. parenteral feeding, broad-spectrum anti-bacterial medication, mechanical ventilation, indwelling central venous as well as bladder catheters, lymphopenia, comorbidities, older age, and corticosteroids are all prevalent in COVID-19 patients admitted to the ICU. The goal of this study was to characterise fungal infection in COVID-19 patients while also investigating the antifungal effects of silver nitrate biosynthesized by *Thymus vulgaris*.

## Discussion

Biogenically synthesized silver nanoparticles have been potent therapeutic agents with prominent antimicrobial properties [51–53]. Although many biogenic nanoparticles have been synthesized still there is need for nanoparticles with precise biological and chemical properties. In this regard use of plant material in the formation of nanoparticles is getting more and more attention [54, 55]. Many scientists used the plant material (bark, leaves, stem, roots, leaves) in the successful synthesis of silver nanoparticles [56–59]. In this study, the hydrothermal method has been adopted for the biogenic synthesis of silver nanoparticles using *Thymus vulgaris* extract. It is the first time use of *Thymus vulagris* extract in a hydrothermal approach for the synthesis of nanoparticles. The main advantage of this method is that it can produce a bulk amount of silver nanoparticles and the efficiency of this procedure is very much high as compared to other known methods [60–62]. Which may perk from the economical and ecological facet. It is well documented that the use of plant extract in the synthesis of nanoparticles leads to nontoxic properties and natural capping agents [63, 64]. The maximum absorbance of silver nanoparticles has been observed in the UV-visible spectrum at 438 nm to 425 nm due to the surface plasmon vibration in nanoparticles, confirming their synthesis. The results presented here are in good agreement with the previous reports [65–71]. Additionally synthesized T/AgNPs have potential anticandidal properties. FTIR spectrum of T/AgNPs reveals the absorbance peak with variable band intensity as compared to the FTIR spectrum of plant extract [72, 73]. This shifting of absorbance peak may be due to the involvement of biomolecules in the reduction and stabilization of silver ions to silver (0) [74–76]. The HRTEM study indicates that most of the nanoparticles are almost spherical. Very few clusters have been observed that may be size variation or gathering of individual particles. Various sizes of spherical nanoparticles have been also reported in other studies by TEM analysis [54, 77, 78]. It is well known that the biogenically synthesized AgNPs using plants or microorganisms are an effective way the advancement of secure and proficient control approach against resistant bacteria [79–82]. Herein the potential of synthesized T/AgNPs has been determined in anticandidal activity. In this regard forty-six fungal isolates were obtained from different specimens of 60 COVID-19 inpatients; 33 were *Candida* albicans, 10 *Candida non-* albicans isolates (2 *Candida* famata, 4 *Candida* galabrata, 2 *Candida* krusei, and 2 *Candida tropicalis*). It has been reported that bacteria species and fungi are considered co-infections in critically ill patients with COVID-19, which increases its morbidity and mortality15. Numerous strains of *Candida* also have been identified as unique pathogens from COVID-19 patients’ pulmonary specimens (TA, BAL as well as BAS) and recognized as a source of co-infections [83] and the frequency of fungal co-infection was significantly greater than previously reported [83].

It is found that *Candida albicans* isolates were highly virulent as 90.7% of isolates had ALS3 and 76.7% had the Hyr1 gene. Hyr1 and Als3 are hypha-specific genes and the hyphal regulator RAS1 is related to adhesion and increase fungal pathogenicity [84]. The RAS1 was prevalent in 88.4%. Ras signalling is crucial to the integration of environmental cues with morphogenesis and virulence [85]. The SAP4 gene was detected in 88.4 percent of *Candida* isolates in this investigation. Twenty secreted aspartic proteinase (Sap) isoenzymes are responsible for *Candida* sppproteinase’s activity. The sap is encoded by ten genes in *Candida*, SAP1-10. Collagen, keratin, and mucus, among other epithelial and mucosal barrier proteins, degrade in the presence of sap [86].

The *Candida* isolates displayed miconazole and clotrimazole resistance in 51.2% of isolates and 100% of aspergillus isolates. Flucytosine resistance occurred in 76.7%, nystatin resistance in 37.2% and fluconazole resistance in 46.5% of isolates. Many researchers have demonstrated that lower *Candida* susceptibility is strongly related to previous antifungal exposures and an unsuitable prior course of antifungal therapy, according to Aldardeer, N. et al, 2020 [87] and Shan, D.N., 2012 mention similar results to other authors [88, 89]. Fluconazole treatment was discovered to be a potential risk for gene mutation and overexposure, which leads to long term fluconazole-resistant *C*. *parapsilosis* [90]. Plant extracts, which contain biomolecules and plant metabolites including flavonoids, tannins, terpenoids, polyphenols as well as algae and fungal metabolites, have special qualities that allow them to be employed as a reducing and capping agent for the manufacture of pharmaceuticals and stabilisation of nanoparticles (NPs) in a variety of fields, including biomedical, pharmaceutical, and food industries [91]. In the current study, *Thymus vulgaris* derived silver nanoparticles showed a significant antifungal effect with a decrease in the number of resistant isolates than known antifungal fluconazole, miconazole, and nystatin, clotrimazole and flucytosine (P-value <0.05). This finding was in line with Mohammadi, M., et al., who found that Ag NPs manufactured from *thyme* extract have no cytotoxicity at concentrations below 3.5 ppm, making them a viable alternative to Fluconazole for treating superficial fungal infections [38]. Similar results were obtained by Anjugam, M., et al, [92]. The excellent use of plant mediated silver nanoparticles in antibacterial as well as anticancer activities has led to a surge in strong interest among different research groups to employ it, reported by Fahimirad, S., et al, [93].

## Conclusion

In our study stable silver nanoparticles were biosynthesized by an eco-benign, one-pot, clean, and useful approach, using leave extract of *Thymus vulgaris*. Hydrothermal synthesis using plant extract is a reliable and safe method. UV-visible spectroscopy, FT-IR, AFM, and TEM techniques were utilized to characterize synthesized T/AgNPs. and confirmed the well-dispersed nature of nanoparticles. The application of T/AgNPs on virulent strains of C*andida albican* reveals that they are potent antifungal and anti-virulence agents to fight against *Candida albicans*. The synthesised nanoparticles show good antibacterial activity.

The present research work unlocks numerous ways of advanced studies, such as investigating the inhibition mechanism and destruction of the cell wall of *Candida albicans*. Moreover further In vivo cytotoxicity and biocompatibility studies are needed before being used for biomedical applications.
